# Concern for Others Leads to Vicarious Optimism

**DOI:** 10.1177/0956797617737129

**Published:** 2018-01-30

**Authors:** Andreas Kappes, Nadira S. Faber, Guy Kahane, Julian Savulescu, Molly J. Crockett

**Affiliations:** 1 Department of Psychology, City, University of London; 2 Department of Experimental Psychology, University of Oxford; 3 Oxford Uehiro Centre for Practical Ethics, University of Oxford; 4 Department of Psychology, Yale University

**Keywords:** optimism, learning bias, altruism, other-regarding preferences, open data, open materials, preregistered

## Abstract

An optimistic learning bias leads people to update their beliefs in response to better-than-expected good news but neglect worse-than-expected bad news. Because evidence suggests that this bias arises from self-concern, we hypothesized that a similar bias may affect beliefs about other people’s futures, to the extent that people care about others. Here, we demonstrated the phenomenon of *vicarious optimism* and showed that it arises from concern for others. Participants predicted the likelihood of unpleasant future events that could happen to either themselves or others. In addition to showing an optimistic learning bias for events affecting themselves, people showed vicarious optimism when learning about events affecting friends and strangers. Vicarious optimism for strangers correlated with generosity toward strangers, and experimentally increasing concern for strangers amplified vicarious optimism for them. These findings suggest that concern for others can bias beliefs about their future welfare and that optimism in learning is not restricted to oneself.

A pervasive bias in learning leads people to change their beliefs about their future more readily when confronted with good news compared with bad news (Sharot & Garrett, 2016). This bias leads to unrealistic optimism, biased self-perceptions, and flawed financial predictions (Eil & Rao, 2011; Kuhnen, 2015; Peysakhovich & Karmarkar, 2016; Russo, Medvec, & Meloy, 1996; Sharot, Korn, & Dolan, 2011). For instance, people readily change their beliefs when learning that their chance of developing cancer during their lifetime is lower than expected but resist updating these same beliefs if they learn that their chance of developing cancer is higher than expected. This learning bias appears to arise from self-enhancing motivations that enable people to develop and maintain positive beliefs about themselves and their future (Greenwald, 1980; Taylor & Brown, 1988; Weinstein, 1980). By contrast, depressed people (who lack self-enhancing motivations) do not show an optimistic learning bias (Garrett et al., 2014; Korn, Sharot, Walter, Heekeren, & Dolan, 2014).

In addition to caring about themselves, people also care about the welfare of others: family, friends, and sometimes even strangers (Engel, 2011; Fehr & Fischbacher, 2003; Hein, Silani, Preuschoff, Batson, & Singer, 2010). Correspondingly, there is evidence that people experience vicarious emotions in response to others’ successes and misfortunes, as reflected in both self-reports of emotions (Goubert et al., 2005; Singer & Klimecki, 2014) and brain activity (Morelli, Sacchet, & Zaki, 2015; Ruff & Fehr, 2014; Zaki, Wager, Singer, Keysers, & Gazzola, 2016). Importantly, the intensity of vicarious emotions is linked to the concern one feels for the other person. For instance, people care more about, and experience more vicarious (empathic) pain, for a fair person compared with an unfair person (Singer et al., 2006) and for in-group compared with out-group members (Hein et al., 2010). Here, we suggest not only that concern for others leads people to vicariously experience negative outcomes for others but also that said concern causes people to bias their learning about others’ negative outcomes—manifesting as *vicarious optimism*. We predicted that participants would show an optimistic bias when learning about the outcomes affecting others they care about, updating their beliefs less in response to bad news compared with good news. We further predicted that increasing concern for others would increase vicarious optimism just as it increases vicarious emotional responses to negative outcomes for others (Hein et al., 2010; Mathur, Richeson, Paice, Muzyka, & Chiao, 2014; Singer et al., 2006).

Previous research suggests that the bias arises from a failure to update beliefs following bad news rather than from an enhanced updating following good news. Updating from good news conforms closely to Bayesian inference, whereas updating from bad news is reduced compared with normative predictions (Eil & Rao, 2011). Moreover, individual differences in optimistic learning track more closely with updating from bad news than good news (Lefebvre, Lebreton, Meyniel, Bourgeois-Gironde, & Palminteri, 2017; Moutsiana et al., 2013; Sharot et al., 2011), and interventions that alter optimistic learning affect updating from bad news but not good news (Sharot, Guitart-Masip, Korn, Chowdhury, & Dolan, 2012; Sharot, Kanai, et al., 2012). This suggests that increasing concern for others may increase vicarious optimism by reducing updating from bad news.

To test whether people indeed optimistically bias their learning about future outcomes for others, we adapted an experimental paradigm previously used to measure learning about the likelihood of negative future events happening to oneself (Kuzmanovic, Jefferson, & Vogeley, 2015; Sharot et al., 2011). In our vicarious optimism task, participants estimated the likelihood of negative future events happening to another person (see Fig. 1). During the task, participants first estimated the likelihood of a negative future event happening to a target person, specified as either a friend (Study 1) or a stranger (Studies 2–4). They were then informed about the average likelihood of that event actually happening to that person. Good news meant that the average likelihood was lower than the first estimate; bad news meant that the average likelihood was higher than the first estimate. Participants then estimated the likelihood of the event in question a second time. The difference between the first and second estimates indicated their level of belief updating. Using this paradigm, we tested whether participants showed vicarious optimism for friends and strangers.

**Fig. 1.**
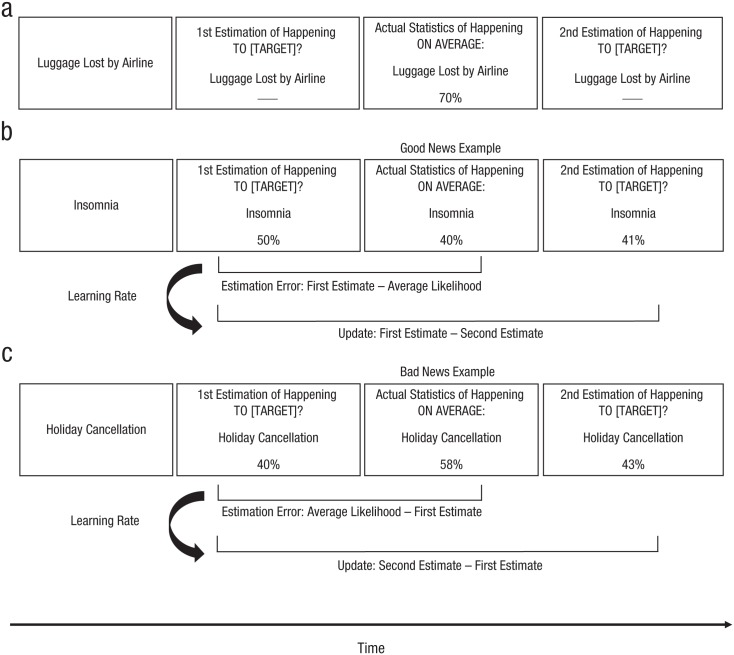
Vicarious optimism task. On each trial (a), participants imagined a negative event happening to a target individual (friend or stranger), estimated the likelihood of the event happening to the target, learned about the average likelihood for that event, and finally reestimated the likelihood. A *good-news* event (b) was defined by a first estimate that was higher than the average likelihood. The estimation error was then calculated by subtracting the first estimate from the average likelihood, and the update was calculated by subtracting the first estimate from the second estimate. The learning rate, which indicated how well the estimation error predicted the subsequent update, was the unstandardized regression coefficient indicating the strength of the relationship between the estimation error and the subsequent update. A *bad-news* event (c) was defined by a first estimate that was lower than the average likelihood. The estimation error was then calculated by subtracting the average likelihood from the first estimate, and the update was calculated by subtracting the second from the first estimate. Again, the learning rate indicated how well the estimation error predicted the subsequent update.

Following past work (Sharot et al., 2011), we measured participants’ optimistic learning bias using (a) the difference in learning rate for good and bad news and (b) the difference in updating for good and bad news. Learning rates indicate the strength of the relationship between the estimation error (i.e., the difference between estimated and average likelihood) and the subsequent belief update on a trial-by-trial basis; the higher the learning rate, the more participants changed their beliefs in line with the conflicting evidence they received. An optimistic bias in learning rates is indicated by stronger learning rates for good versus bad news. Updating, the difference between first and second estimates, indicates the amount a belief is updated when a person receives conflicting information. An optimistic bias in updating indicates that participants changed their beliefs more following good versus bad news. Given that both indicators were associated with the same pattern of results, we report the learning rate results here and the update bias results in the Supplemental Material available online.

In all of our analyses, we controlled for potential differences in estimation errors for good versus bad news to ensure that differences in learning for good versus bad news did not reflect differences in the initial estimates (i.e., prior beliefs) between conditions or mean estimation errors. This also excluded the possibility that the results presented below were a statistical artifact resulting from skewed base rates at the individual level (Garrett & Sharot, 2017). Detailed descriptions of our planned analyses as well as the full set of materials, including all additional measures, can be found at the Open Science Framework (osf.io/6b4ag).

## Study 1: Vicarious Optimism in Learning About Friends

To test whether vicarious optimism in learning exists, we examined whether participants exhibited an optimistic learning bias for their friends as well as for themselves. We therefore had participants complete learning tasks that concerned outcomes for themselves and one of their friends.

### Method

#### Participants

We recruited 83 participants (male = 40, female = 43; mean age = 35.96 years, *SD* = 1.49) online via Amazon Mechanical Turk (MTurk) and compensated them for their time in line with the U.S. minimum wage. We excluded 12 participants with a score higher than 12 on the Beck Depression Inventory (BDI; Beck, Steer, Ball, & Ranieri, 1996; a score indicating mild depression) because depression has been shown to eliminate the optimistic learning bias (Garrett et al., 2014; Korn et al., 2014). Participants had to provide at least four valid updates after good news and four valid updates after bad news, which ensured the reliability of our measures; 3 participants failed to reach these numbers.

We used G*Power 3.1 (Faul, Erdfelder, Buchner, & Lang, 2009) to calculate desired sample sizes. We based our power analysis on previous research on the optimistic learning bias (Garrett & Sharot, 2014; Garrett et al., 2014; Korn et al., 2014; Kuzmanovic et al., 2015; Moutsiana et al., 2013; Sharot, Guitart-Masip, et al., 2012; Sharot, Kanai, et al., 2012; Sharot et al., 2011), which found an average effect size (Cohen’s *d*) of valence (good news vs. bad news) on learning of 0.9. We expected the valence effect for a friend to be less strong than for the self and, hence, estimated a small to medium effect size of 0.4. To achieve a power of .90, we needed a sample of 67 participants. We expected that about 15% of the participants would have a BDI score higher than 12 given our previous experience with MTurk participants. Hence, we aimed for 85 participants to ensure a final sample of about 70 after excluding ineligible participants.

#### Procedure and materials

All participants completed the vicarious optimism task for themselves and a friend. The order of conditions was randomized so that half the participants started with the self condition and the other half started with the friend condition. In each condition (self and friend), participants saw 30 different short descriptions of negative life events (e.g., luggage lost by airline) presented in a random order. To rule out the possibility that observed differences in learning from good versus bad news could reflect a statistical artifact (Garrett & Sharot, 2017), we used credible feedback for participants’ estimations, taken from sources such as the Office for National Statistics (Sharot, Guitart-Masip, et al., 2012). Second, we ensured that base rates used for feedback were normally distributed around the midpoint of the scale we used, such that there was equal room for providing over- and underestimations. The lists of events did not include very rare or very common events, and participants were told that the average likelihood was never lower than 3% or higher than 77%. The first list had a mean base rate of 36.5 (*SD* = 17.71, minimum = 10, maximum = 70), and the second list had a mean base rate of 35.0 (*SD* = 17.23, minimum = 10, maximum = 70). Using the Shapiro-Wilk test of normality, we found that for both lists, the distributions of base rates did not significantly differ from normal distributions, *p*s < .16.

In the self condition, participants were asked to estimate the likelihood of negative life events happening to themselves. In the friend condition, participants were asked to estimate the likelihood of negative life events happening to a friend with the same gender, age, and ethnicity as themselves (participants were asked to enter the name of the friend into a text box prior to starting the trials). The order of conditions did not affect the results.

On each trial, a negative life event was presented, and participants were asked to imagine this event happening to themselves or their friend. Then, when the words “Estimation of happening?” appeared on the screen, participants were prompted to enter the percentage likelihood of this event happening to themselves or their friend at some point in their lifetime, with higher numbers indicating that it was more likely that the event would happen. Participants were told that they would then see the actual average likelihood of this event happening to someone with similar demographic criteria to themselves or their friend. Finally, participants were asked to again indicate the percentage likelihood of the negative event happening to themselves or their friend; they were instructed to indicate what they thought at that moment, regardless of what they entered previously.

Participants initially completed a practice session with three trials. Participants then estimated and reestimated the likelihood of 30 different negative life events happening to themselves or their friend (see Fig. 1). We used two different lists of 30 events (Lists A and B). For half the participants, List A was presented in the self condition and List B was presented in the friend condition; for the other half, the assignment of list to condition was reversed. The pairing of list with condition did not affect the results.

### Results

**Fig. 2.**
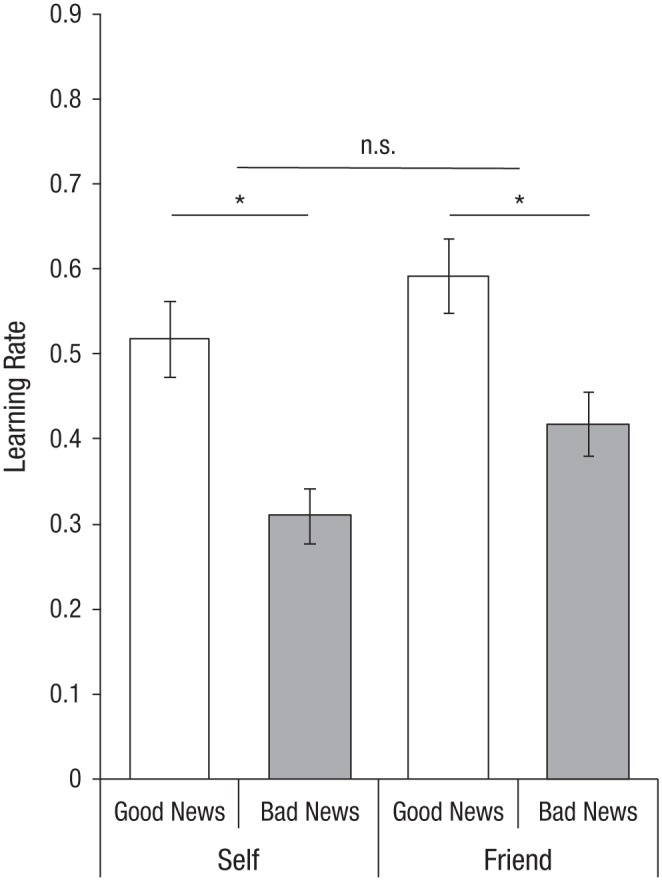
Results from Study 1 (*N* = 68): mean learning rate as a function of whether participants received good news versus bad news, separately for the self and friend conditions. The learning rate is the unstandardized regression coefficient indicating the strength of the relationship between the estimation error and the subsequent update. Error bars represent standard errors of the mean. Asterisks indicate significant differences between conditions (*p* < .05).

To test for vicarious optimism in learning, we examined whether participants exhibited an optimistic learning bias for their friends as well as for themselves. We examined the learning rate (the unstandardized regression coefficient indicating the strength of the relationship between the estimation error and the subsequent update) for good and bad news for the self and a friend using repeated measures analyses of variance, controlling for the differences in estimation errors between good and bad news for both conditions (see Fig. 2).

Across both good- and bad-news conditions, participants more readily changed their beliefs about their friends (*M* = 0.51, *SE* = 0.03) than their beliefs about themselves (*M* = 0.41, *SE* = 0.03), as indicated by a main effect of target, *F*(1, 66) = 8.84, *p* = .004, η_*p*_^2^ = .12. Nevertheless, participants showed optimistic learning not only for themselves but also for their friends, indicated by a main effect of valence, *F*(1, 66) = 25.39, *p* < .001, η_*p*_^2^ = .29, with higher learning rates for good news (*M* = 0.55, *SE* = 0.04) than for bad news (*M* = 0.36, *SE* = 0.03). Simple-effects analyses showed that the bias in learning rates was significantly greater than zero for both the self condition, *t*(67) = 4.68, *p* < .001, 95% confidence interval (CI) = [0.11, 0.30], and the friend condition, *t*(67) = 3.19, *p* = .002, 95% CI = [0.07, 0.28]. The interaction between valence and target was not significant, *F*(1, 66) = 0.24, *p* = .62, indicating that the size of the optimistic learning bias was similar for the self and friend conditions. Overall, 69.1% of participants showed an optimistic bias in their learning rates for themselves (i.e., the difference between learning from good news and learning from bad news was greater than zero), and 63.2% showed an optimistic bias for their friend; there was a strong correlation between both indices, *r*(68) = .58, *p* < .001 (see Fig. S3a in the Supplemental Material). Having found that participants indeed showed vicarious optimism for another person they care about, we next investigated the causal role of concern for others in vicarious optimism.

## Study 2: Vicarious Optimism Is Greater for Identifiable Strangers

In Studies 2a and 2b, we manipulated the concern participants had for a stranger and then measured vicarious optimism for that stranger. We utilized the identifiability effect, whereby people have much greater concern for an identifiable person than for an unidentifiable person (Kogut & Ritov, 2005; Loewenstein, Small, & Strnad, 2005). In the identifiable-stranger condition, we presented participants with a description of a person identified with a name and picture. In the unidentifiable-stranger condition, we presented a separate group of participants with the same description without the name and the picture. We hypothesized that participants would show greater vicarious optimism for an identifiable stranger than an unidentifiable stranger. We were also interested in how the magnitude of vicarious optimism for an identifiable stranger would compare with that for a friend: Would people treat strangers more like friends, in terms of their vicarious optimism, when those strangers are identifiable?

### Method

#### Participants

In Study 2a, for our main test (the difference of the valence effect between the identifiable-stranger and unidentifiable-stranger conditions), we expected a small to medium difference (*d* = 0.4). We needed 100 participants per condition for a power of .80. Again, we expected that about 15% of the participants on MTurk would have a BDI score higher than 12. Hence, we recruited 240 participants (mean age = 28.31 years, *SD* = 0.51; male = 122, female = 117, other = 1). We excluded 51 participants with a BDI score higher than 12 and a further 16 participants who failed to provide at least four valid positive and negative updates on the learning task.

For Study 2b, we preregistered the hypothesis, sampling procedures, and analysis plan (https://osf.io/a5bmw/). To obtain .90 power to detect the effect size in the difference between identifiable and unidentifiable strangers found in Study 2a, we aimed to recruit 470 participants after exclusion of participants with a BDI score higher than 12. Hence, we collected participants until we obtained 470 useable participants (mean age = 38.57 years [2 missing], *SD* = 12.51; male = 205, female = 265; see the preregistered material for further details). Note that we preregistered a different statistical analysis plan from that reported below. We report the preregistered analyses in the Supplemental Material, which produced the same results as reported here.

#### Procedure

In Study 2a, the learning paradigm contained as within-subjects factors a friend condition (identical to Study 1) and a stranger condition. Participants completed these conditions in random order (condition order did not affect the results). For the stranger condition, participants were further randomly assigned to complete the task for either an identifiable stranger or an unidentifiable stranger. Thus, friend versus stranger was manipulated within subjects, and identifiable versus unidentifiable stranger was manipulated between subjects. In the identifiable-stranger condition, participants saw a photograph of an American woman along with a name and brief description, such as, “The stranger is Laura, a 35-year-old female American, pictured below.” Participants saw one of three different women, which ensured that our manipulation did not depend on a specific stimulus set. In the unidentifiable-stranger condition, participants read the same description of the stranger but without the name and the picture: “The stranger is a 35-year-old female American.” In Study 2b, participants were randomly assigned to complete the vicarious optimism task for either an identifiable or an unidentifiable stranger (manipulated between subjects).

### Results

**Fig. 3.**
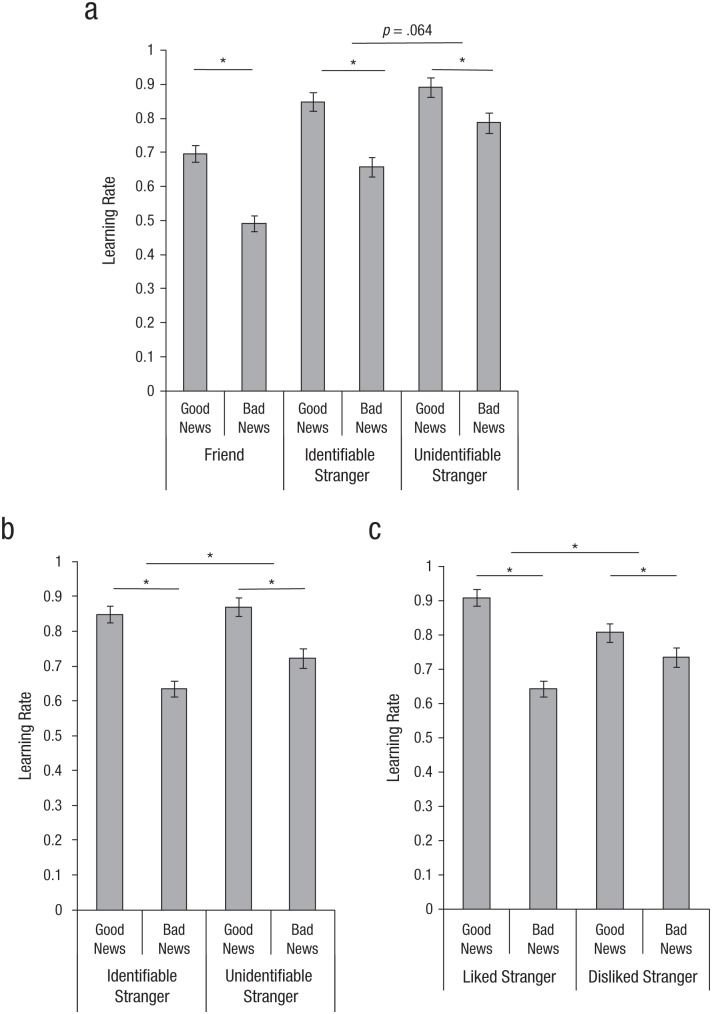
Results from (a) Study 2a (*N* = 170), (b) Study 2b (*N* = 470), and Study 3 (*N* = 285): mean learning rate as a function of whether participants received good versus bad news. Results are shown separately for the friend, identifiable-stranger, and unidentifiable-stranger conditions (Study 2a); the identifiable- and unidentifiable-stranger conditions (Study 2b); and the liked- and disliked-stranger conditions (Study 3). The learning rate is the unstandardized regression coefficient indicating the strength of the relationship between the estimation error and the subsequent update. Error bars represent standard errors of the mean. Asterisks indicate significant differences between conditions (*p* < .05).

Participants’ beliefs about their friends were more resistant to new information than their beliefs about strangers—main effect of target: *F*(1, 167) = 62.49, *p* < .001, η_*p*_^2^ = .27. We found a three-way interaction between valence, target (friend vs. stranger), and identifiability (identifiable vs. unidentifiable stranger) on learning rates, *F*(1, 167) = 8.24, *p* = .005, η_*p*_^2^ = .05, supporting our predictions. Participants showed significantly greater vicarious optimism for friends than for unidentifiable strangers, *F*(1, 77) = 7.58, *p* = .007, but this difference vanished when the stranger was identifiable, *F*(1, 89) = 0.92, *p* = .34. We also found tentative support for the predicted two-way interaction between valence and identifiability, *F*(1, 167) = 3.25, *p* = .073 (see Fig. 3a). Specifically, participants tended to show stronger vicarious optimism for the identifiable stranger compared with the unidentifiable stranger. In line with this pattern, 74.4% of the participants showed an optimistic bias in their learning rates for their friend, 68.7% showed an optimistic bias for an identifiable stranger, and 59.2% showed an optimistic bias for an unidentifiable stranger. Identifiability increased vicarious optimism specifically by reducing updating from bad news, *t*(168) = 3.19, *p* = .002; identifiability did not affect updating from good news, *t*(168) = 1.11, *p* = .27.

Because the predicted interaction between valence and identifiability was only at trend level, we conducted a preregistered replication study using a larger sample on the basis of the effect size found in Study 2a (osf.io/a5bmw/). Here, the interaction between valence and identifiability was significant, *F*(1, 467) = 4.38, *p* = .037, η_*p*_^2^ = .009. As predicted, participants showed greater vicarious optimism, as indicated by biased learning rates, for the identifiable stranger than for the unidentifiable stranger (see Fig. 3b). Consistent with this finding, a total of 69.9% of the participants showed an optimistic bias in their learning rates for an identifiable stranger, and 59.2% for an unidentifiable stranger. As in Study 2a, identifiability reduced updating from bad news, *t*(468) = 3.17, *p* = .002, but did not affect updating from good news, *t*(168) = 0.89, *p* = .37. Thus, increasing concern for a stranger by making the stranger identifiable increased vicarious optimism for that stranger.

One possible alternative explanation for our finding that identifiability increases vicarious optimism is that participants may have felt that they had more information about the identifiable person (Moore & Small, 2007) rather than increased concern for that person. Study 3 was designed to rule out this alternative explanation.

## Study 3: Vicarious Optimism Is Greater for Likable Strangers

People show more concern for those they like compared with those they do not like (Fiske, Cuddy, & Glick, 2007). Thus, we predicted that manipulating likability by providing information about others’ moral decisions (Siegel, Crockett, & Dolan, 2017) would increase vicarious optimism.^1^ Participants read about two strangers who participated in a “Shock Study.” Both strangers received £20 and were asked how much they would be willing to pay to prevent another person from receiving painful electric shocks. The “liked” stranger gives £20.00, whereas the “disliked” stranger gives only £1.96. Consistent with previous findings (Siegel et al., 2017), a pilot study showed that participants rated the liked stranger higher in likability, but not in health or intelligence, than the disliked stranger. Hence, differences in vicarious optimism between the conditions cannot be explained by different perceptions of health or intelligence for the liked versus disliked strangers, since they did not differ on these dimensions.

### Method

#### Participants

As in Study 2b, we preregistered our hypothesis, methods, participant recruitment strategy, and analysis before running the studies and analyzing the data (osf.io/zmf4c). Our goal was to obtain .90 power to detect a small effect size (*f*  ) of .1 at the standard .05 alpha error probability; therefore, we aimed to recruit 280 participants with a BDI score lower than 12 (see the preregistered material for further details). We ended up recruiting 285 participants (mean age = 36.57 years, *SD* = 12.09; male = 132, female = 150, other = 3) via Prolific, a crowdsourcing site similar to Amazon MTurk (Peer, Brandimarte, Samat, & Acquisti, 2017). The study had a within-subjects design in which all participants did the learning task for the liked and disliked strangers in randomized order.

#### Procedure and materials

All participants read descriptions of two strangers, presented in randomized order. Person Y was the liked stranger, whereas Person X was the disliked stranger. In particular, participants read,

Imagine Person X [Y] is participating in an experiment. The experimenter gives Person X [Y] £20. The experimenter then asks how much of this money they would be willing to pay to prevent another person from getting 10 painful electric shocks. Person X [Y] responds that they are willing to pay £1.96 [£20] to prevent another person from getting 10 painful electric shocks.

To ensure that the only difference participants perceived between the strangers was their willingness to help another person to avoid pain, we informed them that “for this Shock Study, we screened participants very carefully so that participants had the same health and intelligence.” Thereafter, we explicitly told participants that this meant that Person X and Person Y have the same level of intelligence and health. Participants finally had to indicate that they understood that both strangers had the same levels of intelligence and health. Then, participants completed the vicarious optimism task for the liked and the disliked strangers in randomized order. For each, they first read the description of the stranger and, thereafter, completed the corresponding vicarious optimism task.

In a pilot study, another set of participants (*N* = 40) rated both strangers (liked and disliked) on likability (likability, generosity, warmth, blameworthiness, morality, friendliness; see the Supplemental Material for factor analyses confirming one general-likability dimension) as well as intelligence and health. Using paired-samples *t* tests, we found a main effect of stranger (liked vs. disliked) on the likability dimension, *t*(39) = 7.79, *p* < .001, but not on intelligence, *t*(39) = 0.75, *p* = .45, or health, *t*(39) = 1.43, *p* = .16. Participants rated the liked stranger as more likable than the disliked stranger, but they rated both as similarly intelligent and healthy.

### Results

We found the expected main effect of valence, *F*(1, 282) = 64.61, *p* < .0001, η_*p*_^2^ = .18; no main effect of person, *F*(1, 282) = .003, *p* = .95; and the predicted interaction effect between valence and likability, *F*(1, 282) = 50.73, *p* < .001, η_*p*_^2^ = .15. Participants showed a stronger bias in learning rates for the liked stranger (mean bias = .26, *SE* = .022) compared with the disliked stranger (mean bias = .07, *SE* = .019). In line with this pattern, 72.6% of the participants showed an optimistic bias in their learning rates for the liked stranger, and 50.5% showed an optimistic bias for the disliked stranger. Consistent with Studies 2a and 2b, likability reduced updating from bad news, *F*(1, 283) = 36.52, *p* < .001. In addition, likability increased updating from good news, *F*(1, 283) = 48.14, *p* < .001. Thus, even though participants’ impressions of the two strangers did not differ in terms of health or intelligence, participants were more optimistic in their learning about the future of the liked stranger who was willing to give all the received money to help a fellow stranger, compared with the disliked stranger, who was willing to give only a meager amount of money to prevent harm to another.

## Study 4: Vicarious Optimism in Learning About Strangers Predicts Altruistic Behavior

In our final study, we examined whether we could predict prosocial behavior from individual levels of vicarious optimism. We first measured participants’ vicarious optimism for a stranger and subsequently their generosity toward similar strangers. Because concern for others predicts prosocial behavior such as donations (Verhaert & Van den Poel, 2011), we reasoned that the stronger participants’ vicarious optimism for an American stranger (indicating the concern they feel for the stranger’s outcomes), the more money they would donate to American strangers in need.

### Method

#### Participants

We expected the valence effect for stranger on learning to be small to medium (*d* = 0.4). To achieve a power of .90, we needed a sample of 67 participants, and for a medium correlation (*r* = .3) between learning bias and charity donation, we needed 80 participants per condition for a power of .80. Hence, we aimed for 100 participants to have a final sample of about 80 after excluding ineligible participants; 95 participants completed the study (mean age = 35.55 years, *SD* = 1.55; male = 43, female = 52). We excluded 18 participants with a BDI score higher than 12 and 4 participants who failed to reach at least four valid positive and negative updates on the learning task.

#### Procedure and materials

Participants completed the vicarious optimism task for themselves and a stranger in randomized order. To ensure that during the stranger task participants were thinking about a single person rather than an undefined group of people, we prompted them to think of a stranger with the same gender, age, and ethnicity as themselves and to come up with and enter a name for the stranger.

After finishing both the self and stranger tasks, participants were given the opportunity to donate to charity. They received a bonus of $1 and then learned about the American Cancer Society, an organization dedicated to helping American people with cancer. Thereafter, participants indicated how many cents out of their $1 bonus—if any—they wanted to donate to the American Cancer Society.

### Results

Again, participants’ beliefs about themselves were more resistant to new information than their beliefs about others—main effect of target, *F*(1, 74) = 45.89, *p* < .001, η_*p*_^2^ = .38. However, people showed optimism for both themselves and strangers—main effect of valence, *F*(1, 74) = 12.75, *p* = .001, η_*p*_^2^ = .15—with learning rates for good news (*M* = 0.67, *SE* = 0.03) being stronger than for bad news (*M* = 0.54, *SE* = 0.03). Simple-effects analyses showed that, across all participants, the bias in learning rates was significantly greater than zero for the self, *t*(75) = 4.5, *p* < .001, 95% CI = [0.10, 0.24], and for the stranger, *t*(75) = 2.61, *p* = .01, 95% CI = [0.02, 0.15]. Nevertheless, we observed pronounced individual differences in the degree of vicarious optimism for strangers, with about half of participants (56.4%) showing an optimistic learning bias and the other half showing a pessimistic learning bias (i.e., a learning rate bias score less than zero). For the self, in contrast, 68.4% showed an optimistic learning bias. The learning bias for the self condition and the learning bias for the stranger condition were not significantly correlated, *r*(76) = .10, *p* = .386 (see Fig. S3b in the Supplemental Material).

**Fig. 4.**
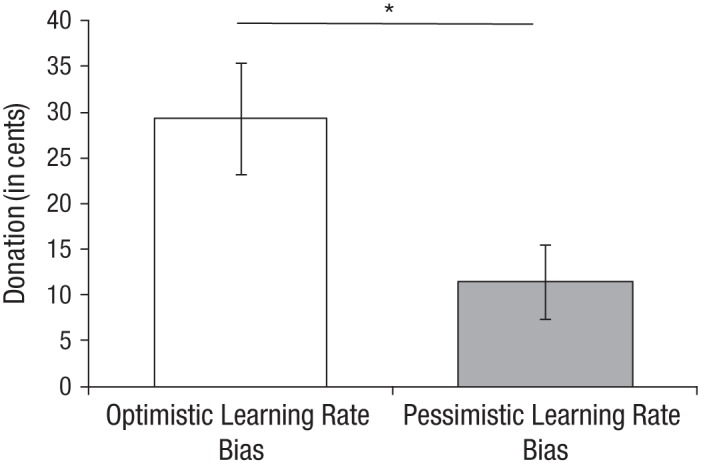
Results from Study 4 (*N* = 76): mean donation amount as a function of participants’ learning rate bias. Error bars represent standard errors of the mean. The asterisk indicates that the difference between conditions was significant (*p* < .05).

Strikingly, we found that participants with an optimistic learning bias for strangers donated on average almost 3 times as much to charity as participants with a pessimistic learning bias for strangers, *t*(74) = 2.26, *p* = .026 (see Fig. 4). Individual differences in the magnitude of vicarious optimism for strangers were positively correlated with donations to charity, *r*(76) = .26, *p* = .02 (see Fig. S4b in the Supplemental Material). This relationship was robust to controlling for the magnitude of the optimistic learning bias for self, age, gender, education, and income, partial *r*(70) = .29, *p* = .012 (see the Supplemental Material for details).

To test whether donations to charity were predicted by reduced updating from bad news, enhanced updating from good news, or both, we regressed donation amount against the learning rates for bad and good news. Age, gender, education, and income were included as control variables. In line with our previous results, results showed that learning from bad news negatively predicted donation amount, β = −0.29, *p* = .02. In addition, learning from good news positively predicted donation amount, β = 0.27, *p* = .03. Thus, our results demonstrate that participants’ vicarious optimism for a stranger is correlated with their altruistic behavior toward similar strangers. Future research is needed to determine the exact mechanism, testing, for instance, whether this relationship between vicarious optimism and prosocial behavior is caused by general individual differences in empathy or target-specific mechanisms such as similarity or shared group membership.

## Discussion

We showed that the concern people feel for others can lead to vicarious optimism. Manipulating the degree of concern for a stranger, by making that person identifiable or more likable, correspondingly increased vicarious optimism for the stranger. Feeling that one knows more about an identifiable compared with an unidentifiable stranger cannot explain these results, because in Study 3, we provided the same amount of information about a liked and a disliked stranger, and participants still showed a substantially stronger bias for the likable stranger, supporting our hypothesis that concern drives vicarious optimism. Furthermore, the degree of vicarious optimism for strangers was predictive of altruistic behavior toward similar strangers. Taken together, these findings suggest that optimism is not restricted to learning about one’s own future outcomes and that vicarious optimism indexes concern for others.

What makes participants more vicariously optimistic in their learning for identifiable and liked strangers, relative to unidentifiable and disliked strangers: enhanced updating from good news or reduced updating from bad news? Consistent with findings showing that manipulations of optimistic learning reduce updating from bad news (Sharot, Guitart-Masip, et al., 2012; Sharot, Kanai, et al., 2012), our results showed that increasing concern for others via identifiability and likability reduced updating from bad news. In addition, we found that likability enhanced updating from good news. These findings suggest that different types of concern might affect vicarious optimism through partially distinct mechanisms, yet more research is needed to systematically test this idea.

Whereas we demonstrated that optimistic learning extends to learning about others, our studies also showed a difference in the way that beliefs about the self and about others are updated, a difference that is rational rather than biased. When comparing learning for the self with learning for others, we found that participants were more reluctant to change their beliefs about themselves than to change their beliefs about a friend or a stranger, indicated by higher learning rates for others compared with the self. Because people have richer and more reliable information about themselves than about others, from a rational (Bayesian) perspective, they should be more reluctant to change their beliefs about themselves than about others because the former are more precise than the latter (Nassar et al., 2012). Thus, learning about others, as captured by our vicarious learning paradigm, was not only characterized by biased processing of good versus bad news but also reflected rational aspects of integrating new information into the beliefs about oneself, friends, and strangers.

Optimism is a self-centered phenomenon in which people underestimate the likelihood of negative future events for themselves compared with others (Weinstein, 1980). Usually, the “other” is defined as a group of average others—an anonymous mass. When past studies asked participants to estimate the likelihood of an event happening to either themselves or the average population, participants did not show a learning bias for the average population (Garrett & Sharot, 2014). These findings are unsurprising given that people typically feel little concern for anonymous groups or anonymous individual strangers (Kogut & Ritov, 2005; Loewenstein et al., 2005). Yet people do care about identifiable others, and we accordingly found that people exhibit an optimistic learning bias for identifiable strangers and, even more markedly, for friends. Our research thereby suggests that optimism in learning is not restricted to oneself. We see not only our own lives through rose-tinted glasses but also the lives of those we care about.

## Supplementary Material

Supplementary material

Supplementary material

## Notes

1. We ran another conceptually similar preregistered study. Preregistered hypotheses and analysis plans, materials, methods, and results for this study can be found at the Open Science Framework (osf.io/n7sfm).

## References

[bibr1-0956797617737129] Beck A. T. Steer R. A. Ball R. Ranieri W. F. (1996). Comparison of Beck Depression Inventories-IA and -II in psychiatric outpatients. Journal of Personality Assessment, 67, 588–597. doi:10.1207/s15327752jpa6703_13 8991972

[bibr2-0956797617737129] Eil D. Rao J. M. (2011). The good news-bad news effect: Asymmetric processing of objective information about yourself. American Economic Journal: Microeconomics, 3, 114–138. doi:10.1257/mic.3.2.114

[bibr3-0956797617737129] Engel C. (2011). Dictator games: A meta study. Experimental Economics, 14, 583–610. doi:10.1007/s10683-011-9283-7

[bibr4-0956797617737129] Faul F. Erdfelder E. Buchner A. Lang A.-G. (2009). Statistical power analyses using G*Power 3.1: Tests for correlation and regression analyses. Behavior Research Methods, 41, 1149–1160. doi:10.3758/BRM.41.4.1149 19897823

[bibr5-0956797617737129] Fehr E. Fischbacher U. (2003). The nature of human altruism. Nature, 425, 785–791. doi:10.1038/nature02043 14574401

[bibr6-0956797617737129] Fiske S. T. Cuddy A. J. C. Glick P. (2007). Universal dimensions of social cognition: Warmth and competence. Trends in Cognitive Sciences, 11, 77–83. 17188552 10.1016/j.tics.2006.11.005

[bibr7-0956797617737129] Garrett N. Sharot T. (2014). How robust is the optimistic update bias for estimating self-risk and population base rates? PLOS ONE, 9(6), Article e98848. doi:10.1371/journal.pone.0098848 PMC4051586 24914643

[bibr8-0956797617737129] Garrett N. Sharot T. (2017). Optimistic update bias holds firm: Three tests of robustness following Shah et al. Consciousness and Cognition, 50, 12–22. 27836628 10.1016/j.concog.2016.10.013 PMC5380127

[bibr9-0956797617737129] Garrett N. Sharot T. Faulkner P. Korn C. W. Roiser J. P. Dolan R. J. (2014). Losing the rose tinted glasses: Neural substrates of unbiased belief updating in depression. Frontiers in Human Neuroscience, 8, Article 639. doi:10.3389/fnhum.2014.00639 PMC4147849 25221492

[bibr10-0956797617737129] Goubert L. Craig K. D. Vervoort T. Morley S. Sullivan M. J. L. Williams A. C. de C.. . . Crombez G. (2005). Facing others in pain: The effects of empathy. Pain, 118, 285–288. doi:10.1016/j.pain.2005.10.025 16289804

[bibr11-0956797617737129] Greenwald A. G. (1980). The totalitarian ego: Fabrication and revision of personal history. American Psychologist, 35, 603–618. doi:10.1037/0003-066X.35.7.603

[bibr12-0956797617737129] Hein G. Silani G. Preuschoff K. Batson C. D. Singer T. (2010). Neural responses to ingroup and outgroup members’ suffering predict individual differences in costly helping. Neuron, 68, 149–160. doi:10.1016/j.neuron.2010.09.003 20920798

[bibr13-0956797617737129] Kogut T. Ritov I. (2005). The “identified victim” effect: An identified group, or just a single individual? Journal of Behavioral Decision Making, 18, 157–167.

[bibr14-0956797617737129] Korn C. W. Sharot T. Walter H. Heekeren H. R. Dolan R. J. (2014). Depression is related to an absence of optimistically biased belief updating about future life events. Psychological Medicine, 44, 579–592. doi:10.1017/S0033291713001074 23672737 PMC3880066

[bibr15-0956797617737129] Kuhnen C. M. (2015). Asymmetric learning from financial information. The Journal of Finance, 70, 2029–2062. doi:10.1111/jofi.12223

[bibr16-0956797617737129] Kuzmanovic B. Jefferson A. Vogeley K. (2015). Self-specific optimism bias in belief updating is associated with high trait optimism. Journal of Behavioral Decision Making, 28, 281–293. doi:10.1002/bdm.1849

[bibr17-0956797617737129] Lefebvre G. Lebreton M. Meyniel F. Bourgeois-Gironde S. Palminteri S. (2017). Behavioural and neural characterization of optimistic reinforcement learning. Nature Human Behaviour, 1, Article 0067. doi:10.1038/s41562-017-0067

[bibr18-0956797617737129] Loewenstein G. Small D. Strnad J. (2005). Statistical, identifiable and iconic victims and perpetrators. SSRN. Retrieved from http://papers.ssrn.com/abstract=678281

[bibr19-0956797617737129] Mathur V. A. Richeson J. A. Paice J. A. Muzyka M. Chiao J. Y. (2014). Racial bias in pain perception and response: Experimental examination of automatic and deliberate processes. The Journal of Pain, 15, 476–484. doi:10.1016/j.jpain.2014.01.488 24462976 PMC4011980

[bibr20-0956797617737129] Moore D. A. Small D. A. (2007). Error and bias in comparative judgment: On being both better and worse than we think we are. Journal of Personality and Social Psychology, 92, 972–989. doi:10.1037/0022-3514.92.6.972 17547483

[bibr21-0956797617737129] Morelli S. A. Sacchet M. D. Zaki J. (2015). Common and distinct neural correlates of personal and vicarious reward: A quantitative meta-analysis. NeuroImage, 112, 244–253. doi:10.1016/j.neuroimage.2014.12.056 25554428 PMC4408229

[bibr22-0956797617737129] Moutsiana C. Garrett N. Clarke R. C. Lotto R. B. Blakemore S.-J. Sharot T. (2013). Human development of the ability to learn from bad news. Proceedings of the National Academy of Sciences, USA, 110, 16396–16401. 10.1073/pnas.1305631110 PMC3799330 24019466

[bibr23-0956797617737129] Nassar M. R. Rumsey K. M. Wilson R. C. Parikh K. Heasly B. Gold J. I. (2012). Rational regulation of learning dynamics by pupil-linked arousal systems. Nature Neuroscience, 15, 1040–1046. doi:10.1038/nn.3130 22660479 PMC3386464

[bibr24-0956797617737129] Peer E. Brandimarte L. Samat S. Acquisti A. (2017). Beyond the Turk: Alternative platforms for crowdsourcing behavioral research. Journal of Experimental Social Psychology, 70, 153–163. doi:10.1016/j.jesp.2017.01.006

[bibr25-0956797617737129] Peysakhovich A. Karmarkar U. R. (2016). Asymmetric effects of favorable and unfavorable information on decision making under ambiguity. Management Science, 62, 2163–2178. doi:10.1287/mnsc.2015.2233

[bibr26-0956797617737129] Ruff C. C. Fehr E. (2014). The neurobiology of rewards and values in social decision making. Nature Reviews Neuroscience, 15, 549–562. doi:10.1038/nrn3776 24986556

[bibr27-0956797617737129] Russo J. E. Medvec V. H. Meloy M. G. (1996). The distortion of information during decisions. Organizational Behavior and Human Decision Processes, 66, 102–110. doi:10.1006/obhd.1996.0041

[bibr28-0956797617737129] Sharot T. Garrett N. (2016). Forming beliefs: Why valence matters. Trends in Cognitive Sciences, 20, 25–33. doi:10.1016/j.tics.2015.11.002 26704856

[bibr29-0956797617737129] Sharot T. Guitart-Masip M. Korn C. W. Chowdhury R. Dolan R. J. (2012). How dopamine enhances an optimism bias in humans. Current Biology, 22, 1477–1481. 22795698 10.1016/j.cub.2012.05.053 PMC3424419

[bibr30-0956797617737129] Sharot T. Kanai R. Marston D. Korn C. W. Rees G. Dolan R. J. (2012). Selectively altering belief formation in the human brain. Proceedings of the National Academy of Sciences, USA, 109, 17058–17062. 10.1073/pnas.1205828109 PMC3479523 23011798

[bibr31-0956797617737129] Sharot T. Korn C. W. Dolan R. J. (2011). How unrealistic optimism is maintained in the face of reality. Nature Neuroscience, 14, 1475–1479. 21983684 10.1038/nn.2949 PMC3204264

[bibr32-0956797617737129] Siegel J. Z. Crockett M. J. Dolan R. J. (2017). Inferences about moral character moderate the impact of consequences on blame and praise. Cognition, 167, 201–211. doi:10.1016/j.cognition.2017.05.004 28527671 PMC5552615

[bibr33-0956797617737129] Singer T. Klimecki O. M. (2014). Empathy and compassion. Current Biology, 24, R875–R878. doi:10.1016/j.cub.2014.06.054 25247366

[bibr34-0956797617737129] Singer T. Seymour B. O’Doherty J. P. Stephan K. E. Dolan R. J. Frith C. D. (2006). Empathic neural responses are modulated by the perceived fairness of others. Nature, 439, 466–469. doi:10.1038/nature04271 16421576 PMC2636868

[bibr35-0956797617737129] Taylor S. E. Brown J. D. (1988). Illusion and well-being: A social psychological perspective on mental health. Psychological Bulletin, 103, 193–210. doi:10.1037/0033-2909.103.2.193 3283814

[bibr36-0956797617737129] Verhaert G. A. Van den Poel D. (2011). Empathy as added value in predicting donation behavior. Journal of Business Research, 64, 1288–1295. doi:10.1016/j.jbusres.2010.12.024

[bibr37-0956797617737129] Weinstein N. D. (1980). Unrealistic optimism about future life events. Journal of Personality and Social Psychology, 39, 806–820. doi:10.1037/0022-3514.39.5.806

[bibr38-0956797617737129] Zaki J. Wager T. D. Singer T. Keysers C. Gazzola V. (2016). The anatomy of suffering: Understanding the relationship between nociceptive and empathic pain. Trends in Cognitive Sciences, 20, 249–259. doi:10.1016/j.tics.2016.02.003 26944221 PMC5521249

