# Supplementary material for: Concern for Others Leads to Vicarious Optimism
Source: Psychol Sci. 2018 Jan 30;29(3):379–89. doi: 10.1177/0956797617737129 (PMC5858641; doi:10.1177/0956797617737129)
Supplement: Supplementary material [file KappesSupplemental_Material_rev.pdf]

Supplemental Results

Concern for Others Leads to Vicarious Optimism: Using Belief Update as Dependent Variable

Below we report the results for the learning bias for self and others using update as the dependent variable for each of the studies presented in the main manuscript. For all analyses we controlled for the difference in estimation errors between good and bad news for each condition. Furthermore, for Study 2b, we report the preregistered analyses. Note that we find the same results for the preregistered analysis and the analysis reported in the main manuscript.

Study 1: Vicarious optimism in learning about friends

To test whether vicarious optimism in learning exists, we examined if participants exhibit an optimistic learning bias for their friends as well as for themselves. We examined the update for good and bad news for the self and a friend using Repeated Measures ANOVAs (Figure S1).

Across both good and bad news, participants changed their beliefs more about their friends (M = 7.64, SE = .48) than about themselves (M = 6.72, SE = .42) as indicated by a main effect of target, $F(1,66) = 5.53$, $p = .022$, $\eta^2 partial = .078$. Nevertheless, we found a main effect of valence, $F(1, 66) = 13.43$, $p < .001$, $\eta^2 partial = .17$. Participants updated their beliefs for both self and friend more readily when receiving good news (M = 8.3, SE = .64) compared to bad news (M = 6.09, SE = .52). Simple effects analyses showed a self bias that was significantly different from zero, $t(67) = 3.73$, p < .001, 95% CI [1.44, 4.76]. Importantly, we found vicarious optimism in learning about the friend, indicated by a bias score different from zero, $t(67) = 2.33$, $p = .02$, 95% CI [.32, 4.18]. We did not find an interaction between valence and target, $F(1, 66) = .35$, $p = .55$, $\eta^2 partial = .005$. Participants did not have a significantly stronger bias in updating – the difference between updating from

good news versus bad news – for themselves (*M* = 3.09, *SE* = .83) than for their friends (*M* = 2.25, *SE* = .96).

Figure S1. Participants updated more for good news versus bad news for the self and a friend (Study 1). The difference between the updating biases did not differ from each other.

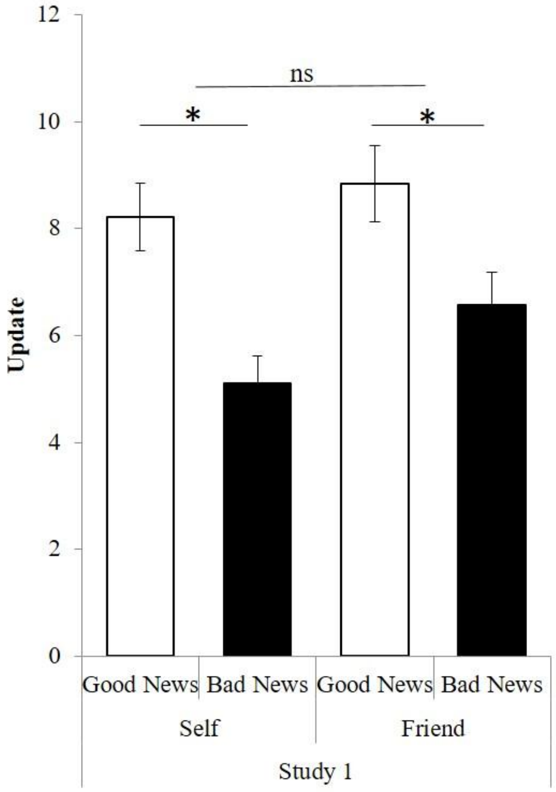

Study 2: Vicarious optimism is greater for identifiable strangers

In Study 2a and b, we manipulated the concern participants had for a stranger and then measured the learning bias for that stranger. We utilized the identifiability effect which reliably shows that people have much greater concern for an identifiable person than for an unidentifiable person.

Using update as dependent variable, valence as within-subject condition, and identifiability as between-subject condition, we found a main effect for valence, $F(1,167) = 9.76$, $p = .002$, $\eta^2 partial = .06$, a main effect for identifiability, $F(1,167) = 4.69$, $p = .03$,

η^2*partial* = .02, and importantly, the predicted interaction effect between valence and stranger, $F(1,167) = 4.19$, $p = .04$. (Figure S2, left). Specifically, participants showed a stronger bias for the identifiable stranger ($M = 2.28$, $SE = .54$) compared to the unidentifiable stranger ($M = .51$, $SE = .73$). In line with this finding, only the bias for the identifiable stranger was significantly greater than zero, $t(90) = 4.2$, $p < .001$, 95% *CI* [1.20, 3.36], but not the bias for the unidentifiable stranger, $t(89) = .69$, $p = .49$, 95% *CI* [-.94, 1.96].

In our preregistered replication (Study 2b), we replicated the difference between identifiable and unidentifiable stranger in updating found in Study 2a. When we preregistered this study, we were not aware of potential confounding effects of estimation errors as reported by Garrett and Sharot (2016). Therefore in our preregistered analysis plan we did not control for the difference in estimation errors between good and bad news as we did for all our analysis in the main manuscript. Here we report the results from the original preregistered analysis plan, for both learning rate and update.

Using learning rates as dependent variable, we found the expected main effect for valence of news (good versus bad), $F(1,468) = 117.50$, $p < .001$, η^2*partial* = .20 – participants had higher learning rates for good versus bad news – a main effect for target (identifiable versus unidentifiable stranger), $F(1, 468) = 7.78$, $p = .006$, η^2*partial* = .016 - participants learned more for the unidentifiable than the identifiable stranger and, importantly, the interaction effect between valence and target, $F(1, 468) = 4.06$, $p = .044$, η^2*partial* = .009. As predicted, participants showed a stronger bias in learning from good news compared to bad news for the identifiable compared to the unidentifiable stranger. Yet both bias scores where significantly greater than zero, *p*s < .001.

Using update as the dependent variable, we found the expected main effect for valence of news (good versus bad), $F(1,468) = 109.20$, $p < .001$, η^2*partial* = .19 – participants update more for good than bad news – a main effect for target (identifiable

versus unidentifiable stranger), $F(1, 468) = 4.78$, $p = .029$, $\eta^2 partial = .01$ - participants updated more for the unidentifiable than the identifiable stranger and, importantly, the interaction effect between valence and target, $F(1, 468) = 5.23$, $p = .023$, $\eta^2 partial = .011$. As predicted, participants showed a stronger bias in updating from good news compared to bad news for the identifiable compared to the unidentifiable stranger. Yet both bias scores where significantly greater than zero, *p*s < .001.

Figure S2. A: Participants showed stronger vicarious optimism for identifiable strangers than for unidentifiable strangers. (Study 2a). B: Participants showed stronger vicarious optimism for an identifiable stranger than for an unidentifiable stranger (Study 2b). C: Participants showed stronger vicarious optimism for a more likeable stranger than a less likeable stranger (Study 3). Error bars in all figures represent standard error of the mean.

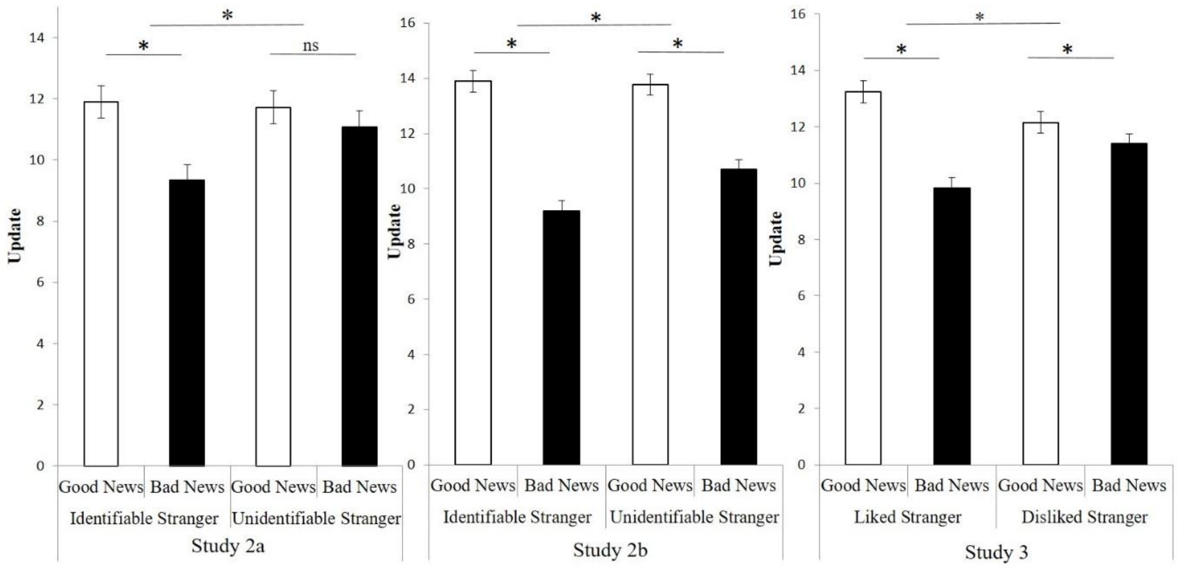

Study 3: Vicarious optimism is greater for identifiable strangers

Pilot study. In order to test if participants indeed perceive the likeability of Person X (i.e., liked stranger) and Person Y (i.e., disliked stranger) differently, we pretested our materials on a separate sample of participants (N = 40). First, participants saw the same descriptions as used in Study 3 (see Methods, Study 3). Thereafter, participants rated Person X and Y on their blameworthiness, generosity, likability, warmth, morality, friendliness, intelligence, and health. These were all the dimensions we measured.

First, we performed a factor analysis to confirm the suspected general likeability dimensions of person perception for the liked and disliked stranger. Scree plot and eigenvalues confirmed one factor, explaining a combined 79.5% (disliked stranger) and 62.86% (liked stranger) of variance. All of the following items load highest on this factor: blameworthiness, generosity, likability, warmth, morality, friendliness. Hence, the factor analysis confirmed one general likeability dimensions of person perception in our data just as a long line of research would have suggested (Fiske, Cuddy, & Glick, 2007). We thereafter formed composite scores for the likeability dimension by computing the mean of the corresponding items. Using paired sample t-tests, we find a main effect of stranger (Person X versus Y) on the likeability dimension, $t(46) = 7.79$, $p < .001$, but not on intelligence, $t(39) = .75$, $p = .45$, or health, $t(39) = 1.43$, $p = .16$. Participants rated the liked stranger as more likeable than the disliked stranger, but rated both as similarly competent.

Next, using update as dependent variable, we performed the preregistered analysis. We found the expected main effect for valence of news (good versus bad), $F(1,282) = 47.52$, $p < .0001$, $\eta^2 partial = .14$ – participants update more for good than bad news – no main effect for stranger (liked versus disliked), $F(1, 282) =.168$, $p = .68$, and, importantly the predicted interaction effect between valence and likeability, $F(1, 282) = 62.24$, $p < .001$, $\eta^2 partial = .18$. Participants showed a stronger bias in updating from good news compared to bad news for the liked stranger compared to the disliked stranger. Both bias scores where significant bigger than zero, *p*s < .001.

Study 4: Vicarious optimism in learning about strangers predicts altruistic behavior

Participants completed the vicarious optimism task for self and a stranger. To ensure that participants were thinking about a single person rather than an undefined group of people, they were prompted to think of a stranger with the same gender, age and ethnicity as themselves and to enter a name for the stranger.

People's beliefs about themselves were more resistant to new information than people's beliefs about strangers (a main effect for target, *F(*1, 74) = 23.08, $p < .001$, η^2*partial* = .23,). However, when examining the updates from good news and bad news for self and stranger, we found a main effect of valence, *F*(1,74) = 6.51, $p = .013$, η^2*partial* = .08, and a an interaction between valence and target, *F*(1, 74) = 4.20, $p = .04$, η^2*partial* = .05. Participants updated more after receiving good news (*M* = 9.68, *SE* = .46) compared to bad news (*M* = 8.01, *SE* = .44). The update bias was stronger for themselves (*M* = 2.4, *SE* = .59) than for the stranger (*M* = .61, *SE* = .59). Finally, while the update bias for self was again significant greater than zero, *t*(75) = 4.39, $p < .001$, 95% *CI* [1.38, 3.61], the update bias for the stranger was not, *t*(75) = 1.29, $p = .20$, 95% *CI* [-.41, 1.93], indicating that participants on average were not biased in their updating about the future of the stranger.

When inspecting the individual differences in the update bias, we see that pronounced individual differences exist. For instance, about half of the participants (56.4%) have an update bias score greater than one (i.e., optimistic update bias) and participants' update bias scores ranged from -13.20 to 14.50 (*M* = .76, *SD* = 5.14). Crucially, we found that participants with an optimistic update bias donated significantly more (*M* = 28.02, *SE* = 6.0) than participants with a pessimistic bias score (*M* = 12.65, *SE* = 4.3). The update bias for the stranger also correlated significantly with the donation amount, *r*(76) = .26, $p = .02$—the higher the update bias, the more participants donated (Figure S4). When controlling for other predictors of donation, such as age, gender, educational level and income level, the correlation between update bias for the stranger and donation increased, *r*(76) = .33, $p = .003$. When controlling additionally for the self-bias, the correlation remained significant, *r*(76) = .32, $p = .004$.

Figure S3. A: The more optimistic the bias in updating about the future of a stranger was, the more participants donated to charity. B: The more optimistic the bias in learning rate about the future of a stranger was, the more participants donated to charity.

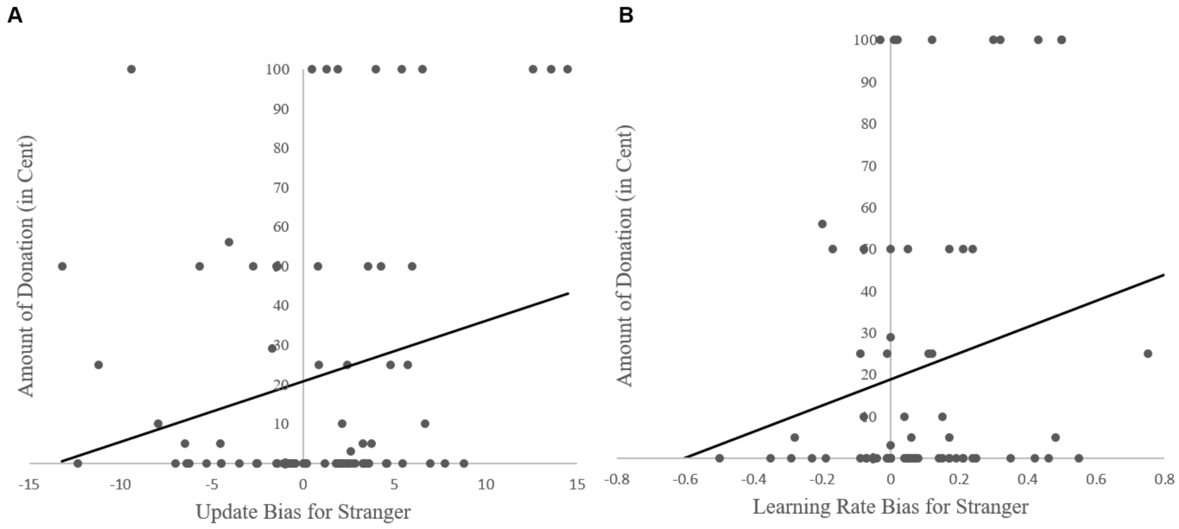

Relationship between Optimism for Self and Others

If vicarious optimism would only be about optimism per se then participants high in optimism for the self would show a similar bias in vicarious optimism, and participants low in optimism for self would also be low in vicarious optimism. However, the learning bias for self and other are now perfectly correlated. While there is a strong correlation between the learning bias for self and friend in Study 1, $r = .58$, $p < .001$ (without one extreme outlier, r = .39, p = .001), we did not find a correlation between the bias for self and unidentifiable stranger in Study 4, $r = .10$, $p = .386$, suggesting that it is “not just about optimism”.

Figure S4 A: The more optimistic the bias in learning about the future for the self, the more optimistic the learning about the future of the stranger (Study 1) B: The bias in learning for the self was not related to the bias in learning for an anonymous stranger (Study 4)

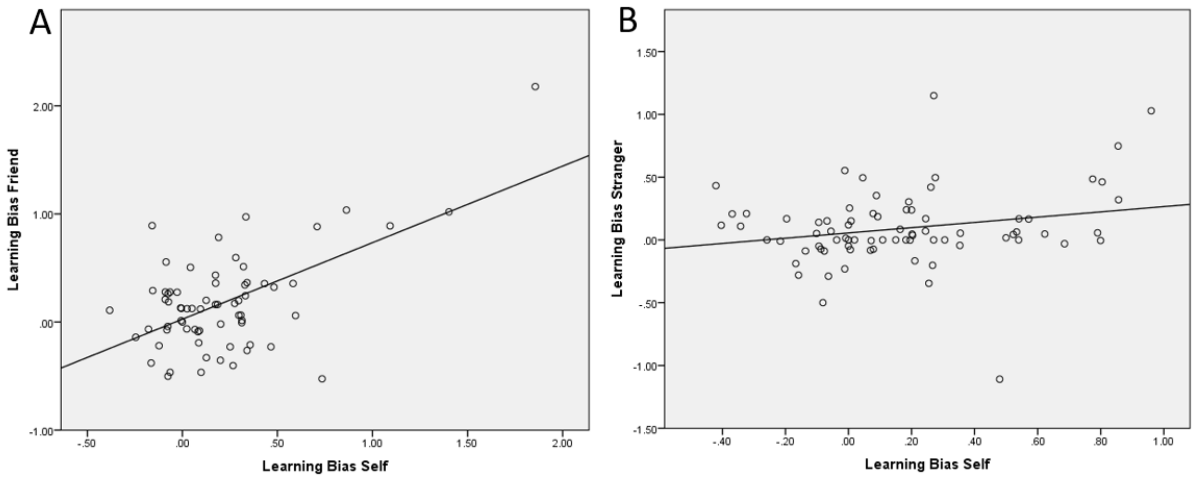

Means and Standard Deviations for Participants' Initial Estimates

Table S1. Displayed are the Means (M) and Standard Error of the Mean (SE) for the initial estimates participants gave before receiving the average likelihood information.

Study	Condition	*M*	*SE*
1	Self	32.16	.76
	Friend	32.02	.89
2a	Friend	32.51	.41
	Identifiable Stranger	33.85	.42
	Unidentifiable Stranger	33.59	.45
2b	Identifiable Stranger	35.07	.32
	Unidentifiable Stranger	35.82	.31
3	Nasty	35.11	.29
	Nice	33.11	.33
4	Self	31.11	.71
	Stranger	33.70	.57

References

Batson, C. D., Eklund, J. H., Chermok, V. L., Hoyt, J. L., & Ortiz, B. G. (2007). An additional antecedent of empathic concern: valuing the welfare of the person in need. *Journal of Personality and Social Psychology*, *93*(1), 65–74. https://doi.org/10.1037/0022-3514.93.1.65

Fiske, S. T., Cuddy, A. J., & Glick, P. (2007). Universal dimensions of social cognition: Warmth and competence. *Trends in Cognitive Sciences*, *11*(2), 77–83.

Singer, T., Seymour, B., O'Doherty, J. P., Stephan, K. E., Dolan, R. J., & Frith, C. D. (2006). Empathic neural responses are modulated by the perceived fairness of others. *Nature*, *439*(7075), 466–469. https://doi.org/10.1038/nature04271
